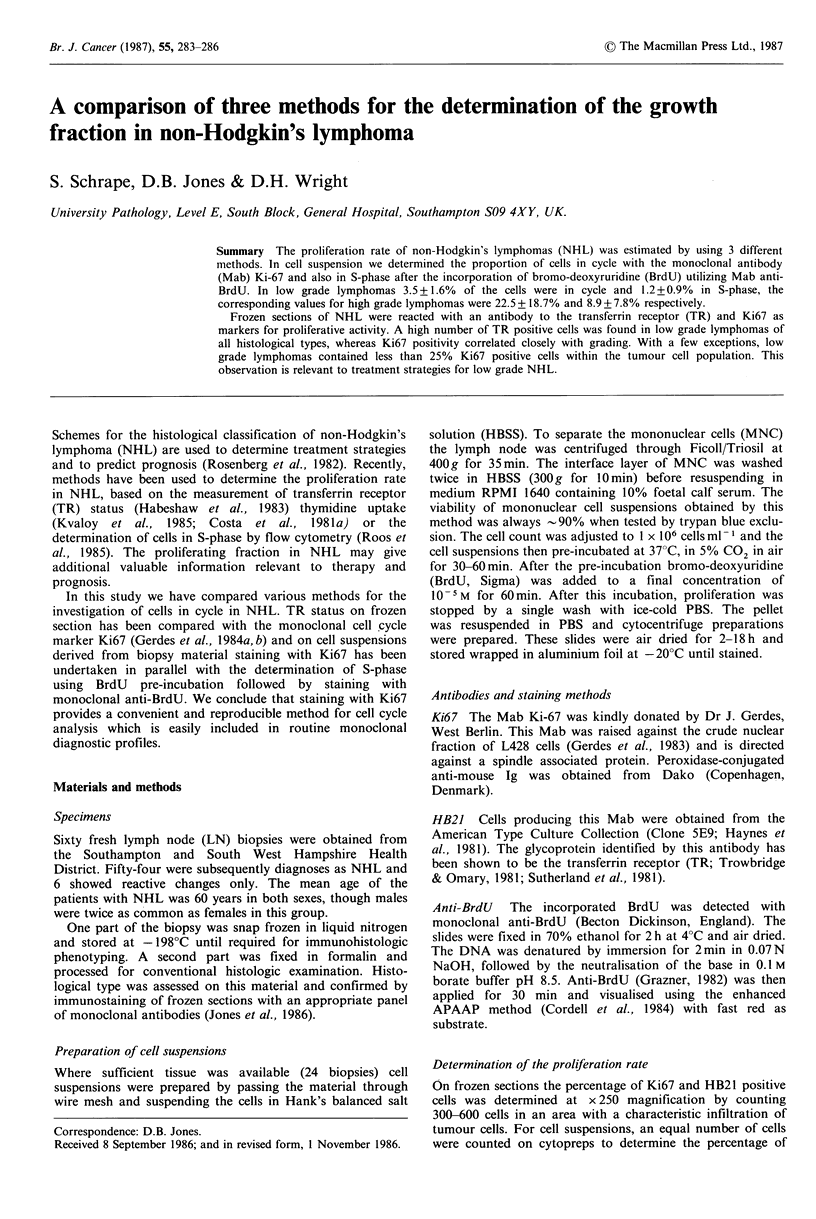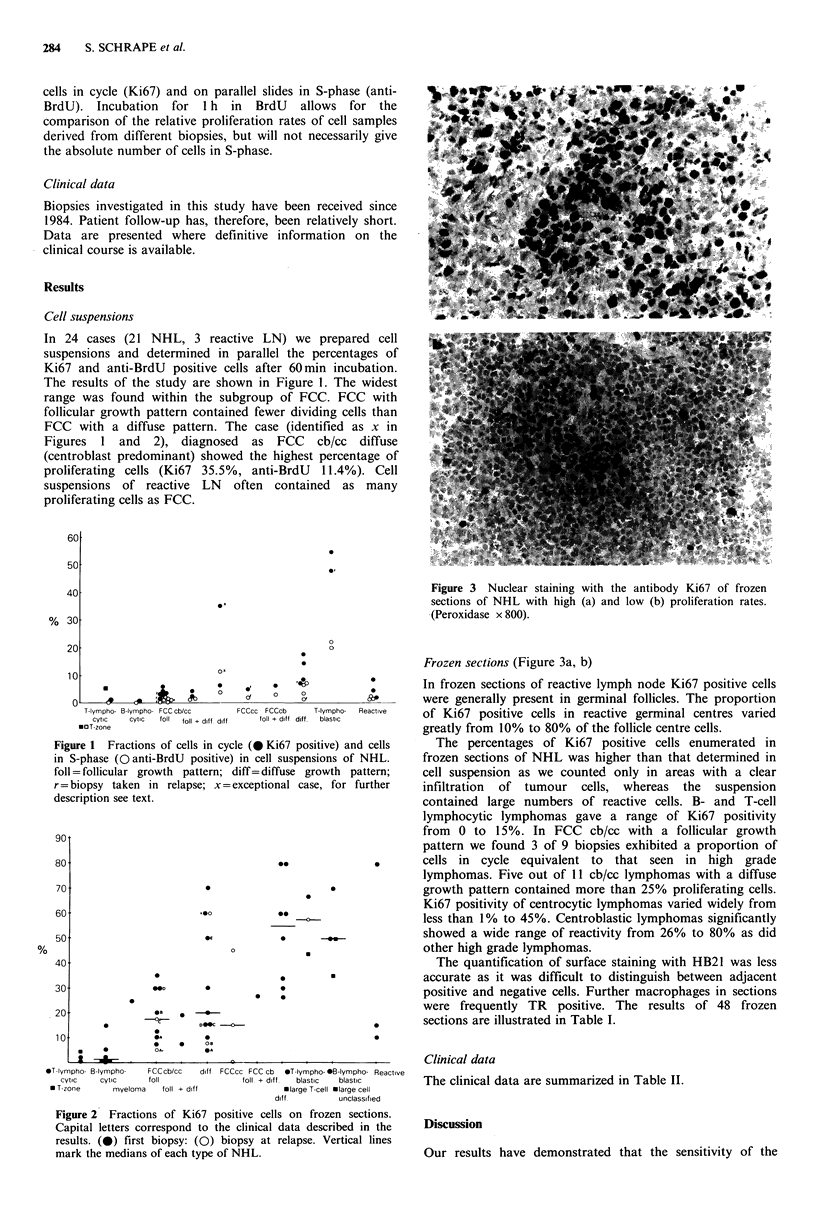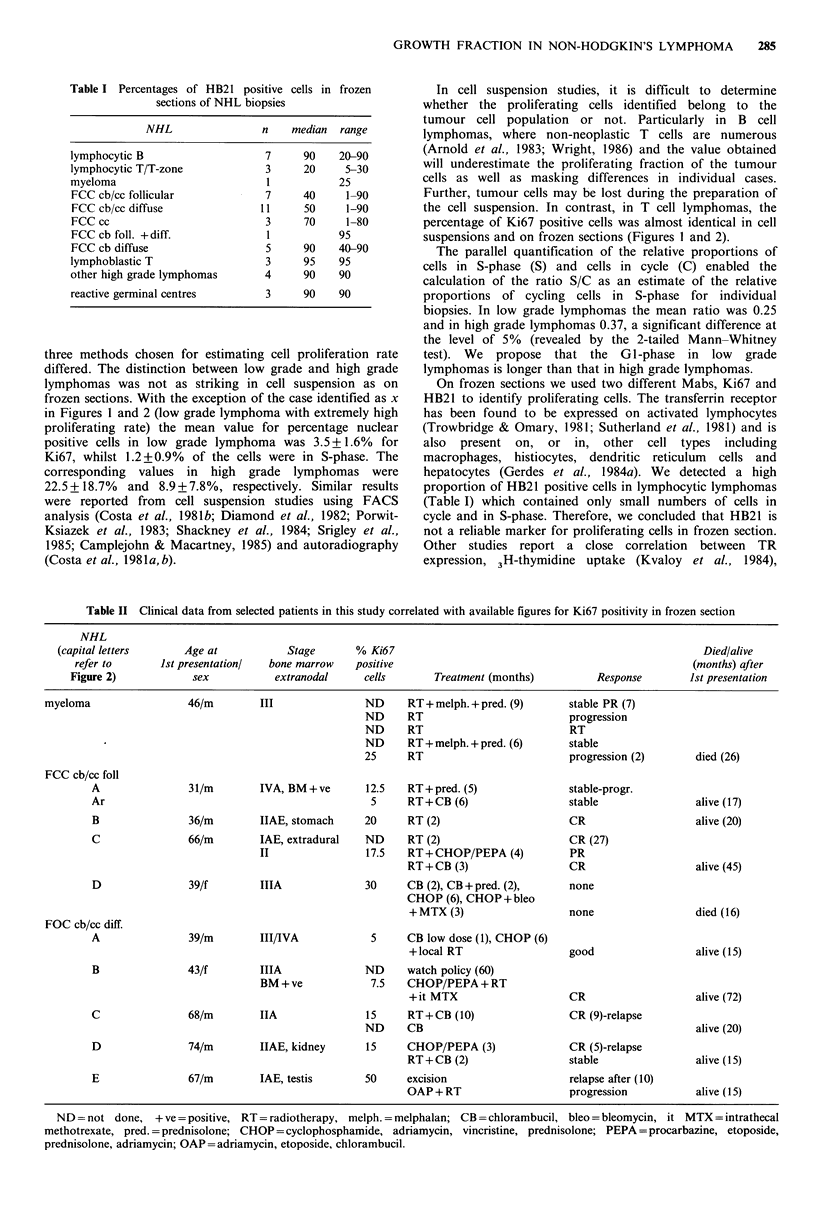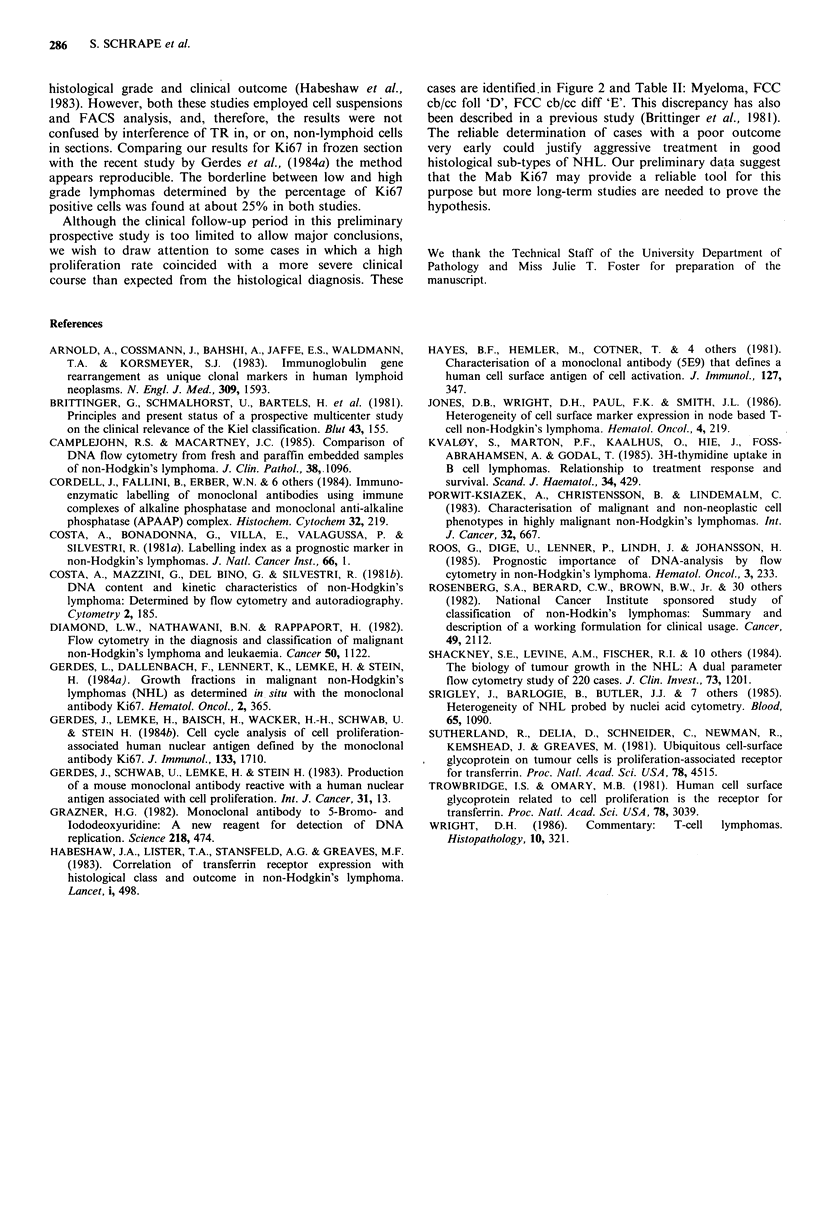# A comparison of three methods for the determination of the growth fraction in non-Hodgkin's lymphoma.

**DOI:** 10.1038/bjc.1987.54

**Published:** 1987-03

**Authors:** S. Schrape, D. B. Jones, D. H. Wright

## Abstract

**Images:**


*Br. J. Cancer* (1987), **55**, 283–286 © The Macmillan Press Ltd., 1987

### A comparison of three methods for the determination of the growth fraction in non-Hodgkin's lymphoma

S. Schrape, D.B. Jones & D.H. Wright

*University Pathology, Level E, South Block, General Hospital, Southampton S09 4XY, UK.*

**Summary** The proliferation rate of non-Hodgkin's lymphomas (NHL) was estimated by using 3 different methods. In cell suspension we determined the proportion of cells in cycle with the monoclonal antibody (Mab) Ki-67 and also in S-phase after the incorporation of bromo-deoxyruridine (BrdU) utilizing Mab anti-BrdU. In low grade lymphomas $3.5 \pm 1.6\%$ of the cells were in cycle and $1.2 \pm 0.9\%$ in S-phase, the corresponding values for high grade lymphomas were $22.5 \pm 18.7\%$ and $8.9 \pm 7.8\%$ respectively.

Frozen sections of NHL were reacted with an antibody to the transferrin receptor (TR) and Ki67 as markers for proliferative activity. A high number of TR positive cells was found in low grade lymphomas of all histological types, whereas Ki67 positivity correlated closely with grading. With a few exceptions, low grade lymphomas contained less than 25% Ki67 positive cells within the tumour cell population. This observation is relevant to treatment strategies for low grade NHL.

Schemes for the histological classification of non-Hodgkin's lymphoma (NHL) are used to determine treatment strategies and to predict prognosis (Rosenberg *et al.*, 1982). Recently, methods have been used to determine the proliferation rate in NHL, based on the measurement of transferrin receptor (TR) status (Habeshaw *et al.*, 1983) thymidine uptake (Kvaloy *et al.*, 1985; Costa *et al.*, 1981*a)* or the determination of cells in S-phase by flow cytometry (Roos *et al.*, 1985). The proliferating fraction in NHL may give additional valuable information relevant to therapy and prognosis.

In this study we have compared various methods for the investigation of cells in cycle in NHL. TR status on frozen section has been compared with the monoclonal cell cycle marker Ki67 (Gerdes *et al.*, 1984*a, b*) and on cell suspensions derived from biopsy material staining with Ki67 has been undertaken in parallel with the determination of S-phase using BrdU pre-incubation followed by staining with monoclonal anti-BrdU. We conclude that staining with Ki67 provides a convenient and reproducible method for cell cycle analysis which is easily included in routine monoclonal diagnostic profiles.

### Materials and methods

*Specimens*

Sixty fresh lymph node (LN) biopsies were obtained from the Southampton and South West Hampshire Health District. Fifty-four were subsequently diagnoses as NHL and 6 showed reactive changes only. The mean age of the patients with NHL was 60 years in both sexes, though males were twice as common as females in this group.

One part of the biopsy was snap frozen in liquid nitrogen and stored at $-198°C$ until required for immunohistologic phenotyping. A second part was fixed in formalin and processed for conventional histologic examination. Histological type was assessed on this material and confirmed by immunostaining of frozen sections with an appropriate panel of monoclonal antibodies (Jones *et al.*, 1986).

*Preparation of cell suspensions*

Where sufficient tissue was available (24 biopsies) cell suspensions were prepared by passing the material through wire mesh and suspending the cells in Hank's balanced salt solution (HBSS). To separate the mononuclear cells (MNC) the lymph node was centrifuged through Ficoll/Triosil at 400 *g* for 35 min. The interface layer of MNC was washed twice in HBSS (300 *g* for 10 min) before resuspending in medium RPMI 1640 containing 10% foetal calf serum. The viability of mononuclear cell suspensions obtained by this method was always $\sim 90\%$ when tested by trypan blue exclusion. The cell count was adjusted to $1 \times 10^6$ cells $ml^{-1}$ and the cell suspensions then pre-incubated at 37°C, in 5% $CO_2$ in air for 30–60 min. After the pre-incubation bromo-deoxyuridine (BrdU, Sigma) was added to a final concentration of $10^{-5}$ M for 60 min. After this incubation, proliferation was stopped by a single wash with ice-cold PBS. The pellet was resuspended in PBS and cytocentrifuge preparations were prepared. These slides were air dried for 2–18 h and stored wrapped in aluminium foil at $-20°C$ until stained.

*Antibodies and staining methods*

*Ki67* The Mab Ki-67 was kindly donated by Dr J. Gerdes, West Berlin. This Mab was raised against the crude nuclear fraction of L428 cells (Gerdes *et al.*, 1983) and is directed against a spindle associated protein. Peroxidase-conjugated anti-mouse Ig was obtained from Dako (Copenhagen, Denmark).

*HB21* Cells producing this Mab were obtained from the American Type Culture Collection (Clone 5E9; Haynes *et al.*, 1981). The glycoprotein identified by this antibody has been shown to be the transferrin receptor (TR; Trowbridge & Omary, 1981; Sutherland *et al.*, 1981).

*Anti-BrdU* The incorporated BrdU was detected with monoclonal anti-BrdU (Becton Dickinson, England). The slides were fixed in 70% ethanol for 2 h at 4°C and air dried. The DNA was denatured by immersion for 2 min in 0.07 N NaOH, followed by the neutralisation of the base in 0.1 M borate buffer pH 8.5. Anti-BrdU (Grazner, 1982) was then applied for 30 min and visualised using the enhanced APAAP method (Cordell *et al.*, 1984) with fast red as substrate.

*Determination of the proliferation rate*

On frozen sections the percentage of Ki67 and HB21 positive cells was determined at $\times 250$ magnification by counting 300–600 cells in an area with a characteristic infiltration of tumour cells. For cell suspensions, an equal number of cells were counted on cytopreps to determine the percentage of

Correspondence: D.B. Jones.
Received 8 September 1986; and in revised form, 1 November 1986.

cells in cycle (Ki67) and on parallel slides in S-phase (anti-BrdU). Incubation for 1 h in BrdU allows for the comparison of the relative proliferation rates of cell samples derived from different biopsies, but will not necessarily give the absolute number of cells in S-phase.

*Clinical data*

Biopsies investigated in this study have been received since 1984. Patient follow-up has, therefore, been relatively short. Data are presented where definitive information on the clinical course is available.

### Results

*Cell suspensions*

In 24 cases (21 NHL, 3 reactive LN) we prepared cell suspensions and determined in parallel the percentages of Ki67 and anti-BrdU positive cells after 60 min incubation. The results of the study are shown in Figure 1. The widest range was found within the subgroup of FCC. FCC with follicular growth pattern contained fewer dividing cells than FCC with a diffuse pattern. The case (identified as $x$ in Figures 1 and 2), diagnosed as FCC cb/cc diffuse (centroblast predominant) showed the highest percentage of proliferating cells (Ki67 35.5%, anti-BrdU 11.4%). Cell suspensions of reactive LN often contained as many proliferating cells as FCC.

**Figure 1** Fractions of cells in cycle (● Ki67 positive) and cells in S-phase (○ anti-BrdU positive) in cell suspensions of NHL. foll = follicular growth pattern; diff = diffuse growth pattern; $r$ = biopsy taken in relapse; $x$ = exceptional case, for further description see text.

**Figure 2** Fractions of Ki67 positive cells on frozen sections. Capital letters correspond to the clinical data described in the results. (●) first biopsy: (○) biopsy at relapse. Vertical lines mark the medians of each type of NHL.

**Figure 3** Nuclear staining with the antibody Ki67 of frozen sections of NHL with high (a) and low (b) proliferation rates. (Peroxidase ×800).

*Frozen sections* (Figure 3a, b)

In frozen sections of reactive lymph node Ki67 positive cells were generally present in germinal follicles. The proportion of Ki67 positive cells in reactive germinal centres varied greatly from 10% to 80% of the follicle centre cells.

The percentages of Ki67 positive cells enumerated in frozen sections of NHL was higher than that determined in cell suspension as we counted only in areas with a clear infiltration of tumour cells, whereas the suspension contained large numbers of reactive cells. B- and T-cell lymphocytic lymphomas gave a range of Ki67 positivity from 0 to 15%. In FCC cb/cc with a follicular growth pattern we found 3 of 9 biopsies exhibited a proportion of cells in cycle equivalent to that seen in high grade lymphomas. Five out of 11 cb/cc lymphomas with a diffuse growth pattern contained more than 25% proliferating cells. Ki67 positivity of centrocytic lymphomas varied widely from less than 1% to 45%. Centroblastic lymphomas significantly showed a wide range of reactivity from 26% to 80% as did other high grade lymphomas.

The quantification of surface staining with HB21 was less accurate as it was difficult to distinguish between adjacent positive and negative cells. Further macrophages in sections were frequently TR positive. The results of 48 frozen sections are illustrated in Table I.

*Clinical data*

The clinical data are summarized in Table II.

### Discussion

Our results have demonstrated that the sensitivity of the

**Table I** Percentages of HB21 positive cells in frozen sections of NHL biopsies

| *NHL* | *n* | *median* | *range* |
|---|---|---|---|
| lymphocytic B | 7 | 90 | 20–90 |
| lymphocytic T/T-zone | 3 | 20 | 5–30 |
| myeloma | 1 | | 25 |
| FCC cb/cc follicular | 7 | 40 | 1–90 |
| FCC cb/cc diffuse | 11 | 50 | 1–90 |
| FCC cc | 3 | 70 | 1–80 |
| FCC cb foll. +diff. | 1 | | 95 |
| FCC cb diffuse | 5 | 90 | 40–90 |
| lymphoblastic T | 3 | 95 | 95 |
| other high grade lymphomas | 4 | 90 | 90 |
| reactive germinal centres | 3 | 90 | 90 |

three methods chosen for estimating cell proliferation rate differed. The distinction between low grade and high grade lymphomas was not as striking in cell suspension as on frozen sections. With the exception of the case identified as *x* in Figures 1 and 2 (low grade lymphoma with extremely high proliferating rate) the mean value for percentage nuclear positive cells in low grade lymphoma was $3.5 \pm 1.6\%$ for Ki67, whilst $1.2 \pm 0.9\%$ of the cells were in S-phase. The corresponding values in high grade lymphomas were $22.5 \pm 18.7\%$ and $8.9 \pm 7.8\%$, respectively. Similar results were reported from cell suspension studies using FACS analysis (Costa *et al.*, 1981*b*; Diamond *et al.*, 1982; Porwit-Ksiazek *et al.*, 1983; Shackney *et al.*, 1984; Srigley *et al.*, 1985; Camplejohn & Macartney, 1985) and autoradiography (Costa *et al.*, 1981*a,b*).

In cell suspension studies, it is difficult to determine whether the proliferating cells identified belong to the tumour cell population or not. Particularly in B cell lymphomas, where non-neoplastic T cells are numerous (Arnold *et al.*, 1983; Wright, 1986) and the value obtained will underestimate the proliferating fraction of the tumour cells as well as masking differences in individual cases. Further, tumour cells may be lost during the preparation of the cell suspension. In contrast, in T cell lymphomas, the percentage of Ki67 positive cells was almost identical in cell suspensions and on frozen sections (Figures 1 and 2).

The parallel quantification of the relative proportions of cells in S-phase (S) and cells in cycle (C) enabled the calculation of the ratio S/C as an estimate of the relative proportions of cycling cells in S-phase for individual biopsies. In low grade lymphomas the mean ratio was 0.25 and in high grade lymphomas 0.37, a significant difference at the level of 5% (revealed by the 2-tailed Mann–Whitney test). We propose that the G1-phase in low grade lymphomas is longer than that in high grade lymphomas.

On frozen sections we used two different Mabs, Ki67 and HB21 to identify proliferating cells. The transferrin receptor has been found to be expressed on activated lymphocytes (Trowbridge & Omary, 1981; Sutherland *et al.*, 1981) and is also present on, or in, other cell types including macrophages, histiocytes, dendritic reticulum cells and hepatocytes (Gerdes *et al.*, 1984*a*). We detected a high proportion of HB21 positive cells in lymphocytic lymphomas (Table I) which contained only small numbers of cells in cycle and in S-phase. Therefore, we concluded that HB21 is not a reliable marker for proliferating cells in frozen section. Other studies report a close correlation between TR expression, $_3$H-thymidine uptake (Kvaloy *et al.*, 1984),

**Table II** Clinical data from selected patients in this study correlated with available figures for Ki67 positivity in frozen section

| *NHL (capital letters refer to* **Figure 2***)* | *Age at 1st presentation/ sex* | *Stage bone marrow extranodal* | *% Ki67 positive cells* | *Treatment* (months) | *Response* | *Died/alive (months) after 1st presentation* |
|---|---|---|---|---|---|---|
| myeloma | 46/m | III | ND | RT+melph.+pred. (9) | stable PR (7) | |
| | | | ND | RT | progression | |
| | | | ND | RT | RT | |
| | | | ND | RT+melph.+pred. (6) | stable | |
| | | | 25 | RT | progression (2) | died (26) |
| FCC cb/cc foll | | | | | | |
| A | 31/m | IVA, BM+ve | 12.5 | RT+pred. (5) | stable-progr. | |
| Ar | | | 5 | RT+CB (6) | stable | alive (17) |
| B | 36/m | IIAE, stomach | 20 | RT (2) | CR | alive (20) |
| C | 66/m | IAE, extradural | ND | RT (2) | CR (27) | |
| | | II | 17.5 | RT+CHOP/PEPA (4) | PR | |
| | | | | RT+CB (3) | CR | alive (45) |
| D | 39/f | IIIA | 30 | CB (2), CB+pred. (2), CHOP (6), CHOP+bleo | none | |
| | | | | +MTX (3) | none | died (16) |
| FOC cb/cc diff. | | | | | | |
| A | 39/m | III/IVA | 5 | CB low dose (1), CHOP (6) +local RT | good | alive (15) |
| B | 43/f | IIIA | ND | watch policy (60) | | |
| | | BM+ve | 7.5 | CHOP/PEPA+RT +it MTX | CR | alive (72) |
| C | 68/m | IIA | 15 | RT+CB (10) | CR (9)-relapse | |
| | | | ND | CB | | alive (20) |
| D | 74/m | IIAE, kidney | 15 | CHOP/PEPA (3) | CR (5)-relapse | |
| | | | | RT+CB (2) | stable | alive (15) |
| E | 67/m | IAE, testis | 50 | excision | relapse after (10) | |
| | | | | OAP+RT | progression | alive (15) |

ND = not done, +ve = positive, RT = radiotherapy, melph. = melphalan; CB = chlorambucil, bleo = bleomycin, it MTX = intrathecal methotrexate, pred. = prednisolone; CHOP = cyclophosphamide, adriamycin, vincristine, prednisolone; PEPA = procarbazine, etoposide, prednisolone, adriamycin; OAP = adriamycin, etoposide, chlorambucil.

histological grade and clinical outcome (Habeshaw *et al.*, 1983). However, both these studies employed cell suspensions and FACS analysis, and, therefore, the results were not confused by interference of TR in, or on, non-lymphoid cells in sections. Comparing our results for Ki67 in frozen section with the recent study by Gerdes *et al.*, (1984*a*) the method appears reproducible. The borderline between low and high grade lymphomas determined by the percentage of Ki67 positive cells was found at about 25% in both studies.

Although the clinical follow-up period in this preliminary prospective study is too limited to allow major conclusions, we wish to draw attention to some cases in which a high proliferation rate coincided with a more severe clinical course than expected from the histological diagnosis. These cases are identified in Figure 2 and Table II: Myeloma, FCC cb/cc foll 'D', FCC cb/cc diff 'E'. This discrepancy has also been described in a previous study (Brittinger *et al.*, 1981). The reliable determination of cases with a poor outcome very early could justify aggressive treatment in good histological sub-types of NHL. Our preliminary data suggest that the Mab Ki67 may provide a reliable tool for this purpose but more long-term studies are needed to prove the hypothesis.

We thank the Technical Staff of the University Department of Pathology and Miss Julie T. Foster for preparation of the manuscript.

### References

ARNOLD, A., COSSMANN, J., BAHSHI, A., JAFFE, E.S., WALDMANN, T.A. & KORSMEYER, S.J. (1983). Immunoglobulin gene rearrangement as unique clonal markers in human lymphoid neoplasms. *N. Engl. J. Med.*, **309**, 1593.

BRITTINGER, G., SCHMALHORST, U., BARTELS, H. *et al.* (1981). Principles and present status of a prospective multicenter study on the clinical relevance of the Kiel classification. *Blut* **43**, 155.

CAMPLEJOHN, R.S. & MACARTNEY, J.C. (1985). Comparison of DNA flow cytometry from fresh and paraffin embedded samples of non-Hodgkin's lymphoma. *J. Clin. Pathol.*, **38**, 1096.

CORDELL, J., FALLINI, B., ERBER, W.N. & 6 others (1984). Immunoenzymatic labelling of monoclonal antibodies using immune complexes of alkaline phosphatase and monoclonal anti-alkaline phosphatase (APAAP) complex. *Histochem. Cytochem* **32**, 219.

COSTA, A., BONADONNA, G., VILLA, E., VALAGUSSA, P. & SILVESTRI, R. (1981*a*). Labelling index as a prognostic marker in non-Hodgkin's lymphomas. *J. Natl. Cancer Inst.*, **66**, 1.

COSTA, A., MAZZINI, G., DEL BINO, G. & SILVESTRI, R. (1981*b*). DNA content and kinetic characteristics of non-Hodgkin's lymphoma: Determined by flow cytometry and autoradiography. *Cytometry* **2**, 185.

DIAMOND, L.W., NATHAWANI, B.N. & RAPPAPORT, H. (1982). Flow cytometry in the diagnosis and classification of malignant non-Hodgkin's lymphoma and leukaemia. *Cancer* **50**, 1122.

GERDES, L., DALLENBACH, F., LENNERT, K., LEMKE, H. & STEIN, H. (1984*a*). Growth fractions in malignant non-Hodgkin's lymphomas (NHL) as determined *in situ* with the monoclonal antibody Ki67. *Hematol. Oncol.*, **2**, 365.

GERDES, J., LEMKE, H., BAISCH, H., WACKER, H.-H., SCHWAB, U. & STEIN H. (1984*b*). Cell cycle analysis of cell proliferation-associated human nuclear antigen defined by the monoclonal antibody Ki67. *J. Immunol.*, **133**, 1710.

GERDES, J., SCHWAB, U., LEMKE, H. & STEIN H. (1983). Production of a mouse monoclonal antibody reactive with a human nuclear antigen associated with cell proliferation. *Int. J. Cancer*, **31**, 13.

GRAZNER, H.G. (1982). Monoclonal antibody to 5-Bromo- and Iododeoxyuridine: A new reagent for detection of DNA replication. *Science* **218**, 474.

HABESHAW, J.A., LISTER, T.A., STANSFELD, A.G. & GREAVES, M.F. (1983). Correlation of transferrin receptor expression with histological class and outcome in non-Hodgkin's lymphoma. *Lancet*, **i**, 498.

HAYES, B.F., HEMLER, M., COTNER, T. & 4 others (1981). Characterisation of a monoclonal antibody (5E9) that defines a human cell surface antigen of cell activation. *J. Immunol.*, **127**, 347.

JONES, D.B., WRIGHT, D.H., PAUL, F.K. & SMITH, J.L. (1986). Heterogeneity of cell surface marker expression in node based T-cell non-Hodgkin's lymphoma. *Hematol. Oncol.*, **4**, 219.

KVALØY, S., MARTON, P.F., KAALHUS, O., HIE, J., FOSS-ABRAHAMSEN, A. & GODAL, T. (1985). 3H-thymidine uptake in B cell lymphomas. Relationship to treatment response and survival. *Scand. J. Haematol.*, **34**, 429.

PORWIT-KSIAZEK, A., CHRISTENSSON, B. & LINDEMALM, C. (1983). Characterisation of malignant and non-neoplastic cell phenotypes in highly malignant non-Hodgkin's lymphomas. *Int. J. Cancer*, **32**, 667.

ROOS, G., DIGE, U., LENNER, P., LINDH, J. & JOHANSSON, H. (1985). Prognostic importance of DNA-analysis by flow cytometry in non-Hodgkin's lymphoma. *Hematol. Oncol.*, **3**, 233.

ROSENBERG, S.A., BERARD, C.W., BROWN, B.W., Jr. & 30 others (1982). National Cancer Institute sponsored study of classification of non-Hodkin's lymphomas: Summary and description of a working formulation for clinical usage. *Cancer*, **49**, 2112.

SHACKNEY, S.E., LEVINE, A.M., FISCHER, R.I. & 10 others (1984). The biology of tumour growth in the NHL: A dual parameter flow cytometry study of 220 cases. *J. Clin. Invest.*, **73**, 1201.

SRIGLEY, J., BARLOGIE, B., BUTLER, J.J. & 7 others (1985). Heterogeneity of NHL probed by nuclei acid cytometry. *Blood*, **65**, 1090.

SUTHERLAND, R., DELIA, D., SCHNEIDER, C., NEWMAN, R., KEMSHEAD, J. & GREAVES, M. (1981). Ubiquitous cell-surface glycoprotein on tumour cells is proliferation-associated receptor for transferrin. *Proc. Natl. Acad. Sci. USA*, **78**, 4515.

TROWBRIDGE, I.S. & OMARY, M.B. (1981). Human cell surface glycoprotein related to cell proliferation is the receptor for transferrin. *Proc. Natl. Acad. Sci. USA*, **78**, 3039.

WRIGHT, D.H. (1986). Commentary: T-cell lymphomas. *Histopathology*, **10**, 321.